# Association of Limb-Girdle muscular dystrophy with multiple sclerosis: A case report

**DOI:** 10.22088/cjim.9.1.96

**Published:** 2018

**Authors:** Mahsa Arzani, Hamed Rezaei, Abdorreza Naser Moghadasi

**Affiliations:** 1 Department of Neurology, Sina Hospital, Tehran University of Medical Sciences, Tehran, Iran.; 2 Department of Urology, Sina Hospital, Tehran University of Medical Sciences, Tehran, Iran.; 3 Sina MS Research Center, Neuroscience Institute, Tehran University of Medical Sciences, Tehran, Iran

**Keywords:** Limb-Girdle Muscular Dystrophy, Multiple Sclerosis, Myopathy

## Abstract

**Background:**

The association of limb-girdle muscular dystrophy (LGMD) with other neurological disorders is uncommon.

**Case presentation:**

We report a 25-year-old female with LGMD who suffered from slowly progressive proximal muscular weakness and atrophy since she was 12 years of age. The patient recently presented with acute loss of left side visual acuity. After evaluation, findings were suggestive of multiple sclerosis.

**Conclusions:**

This is the first report of LGMD in association with MS. The simultaneous occurrence of MS with myopathies may be incidental but there may be a genetic susceptibility for both diseases. This comorbidity may influence the treatment of MS.

Limb-girdle muscular dystrophy (LGMD) denominates a group of diseases that involve proximal muscles in childhood or adulthood. It is sometimes associated with elevated creatine phosphokinase serum levels and indicated by dystrophic changes (regeneration/degeneration) on muscle biopsy. The age of onset, severity, and systemic organ involvement varies among different subtypes. In addition, the signs and symptoms are different among the members of one family (1, 2). We report a known case of LGMD that presented with left optic neuritis during the follow-up. After investigation, a diagnosis of multiple sclerosis (MS) was made based on the magnetic resonance imaging (MRI) findings.

## Case Presentation

The patient was a 25-year-old female born to non-consanguineous parents. She was the second child of a family with one brother and two sisters. Her initial symptom began when she was 12 years old and presented as difficulty in climbing stairs. Her weakness was slowly progressive with the wasting of the proximal leg muscles so that she lost her ability to run. After a few years, she also felt weak in her shoulders and arms but to a lesser degree than her legs. She did not have any sensory or sphincter complaints. Her swallowing was normal. A family history of muscle weakness was positive in her grandmother, but it was not debilitating enough to look for a cause. One of her aunts had MS. Her history was negative for alcohol and smoking. She only took multivitamin pills temporarily. On examination, her body-mass index (BMI) was 23 and she was afebrile with no abnormal findings on physical examination of the skin, mucous, head, and neck. Cardiopulmonary and abdominopelvic examinations showed no abnormalities. She was fully obedient and her mental functions were intact with normal speech. An examination of her cranial nerves showed no facial or bulbar muscle involvement. Muscle weakness and atrophy was symmetrical, prominent in the pelvic and shoulder girdle sparing oculofaciobulbar muscles. 

The muscles had a normal tonus without any evidence of contracture or calf hypertrophy. She had good strength in her axial muscles (5/5). Weakness was more obvious in the lower limbs than in the upper limbs (3/5 and 4/5, respectively). The power of her distal muscles, wrists, and feet was preserved. Her tendon reflexes diminished proportional to their weakness. Plantar reflexes were down bilaterally. Sensory examination was normal. Laboratory tests performed at that time showed an increased level of transaminases (ALT: 72 U/L, AST: 55U/L) and CPK 10000 U/L. Cardiologic consultation revealed no significant cardiac abnormalities. 

**Fig 1:**
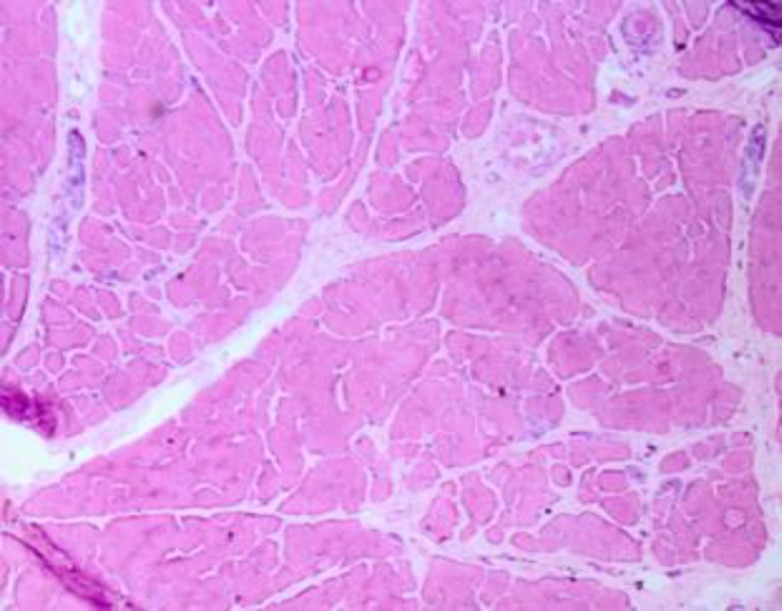
H&E staining: atrophy, some necrotic and regenerative fibers are noted. Endomysial connective tissue is severely increased

**Fig 2:**
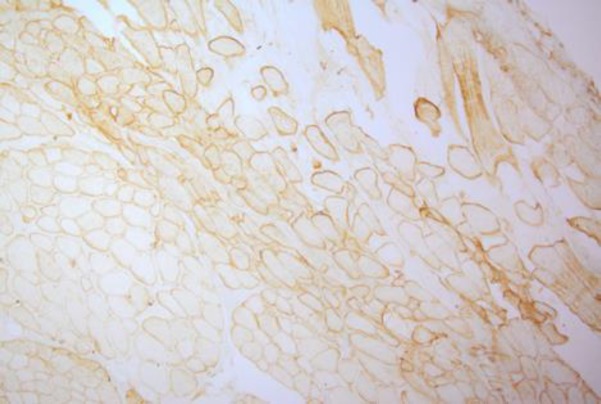
Immunohistochemistry (IHC) shows staining with anti-dystrophin, dysferlin, merosin, and sarcoglycan

The first electromyography (EMG) (2005) showed a myopathic pattern in the lower limbs from which a muscle biopsy was requested but the patient postponed it. Until the patient was 20 years old, her symptoms were progressive; then, she experienced a stabilization of the disease. A biopsy taken in 2011 revealed dystrophic changes (degeneration/ regeneration) with markedly increased endomysial connective tissue; no inflammatory cell infiltration, ragged red fiber, cytoplasmic body and lipid or glycogen accumulation in muscle fibers were seen (fig.1). An immunohistochemistry (IHC) study of sarcolemmal proteins showed sarcolemma labeling of all fibers with DYS1, DYS2, DYS3, alpha and gamma sarcoglycan, dysferlin, merosin, and beta-spectrin (fig.2). 

**Fig 3:**
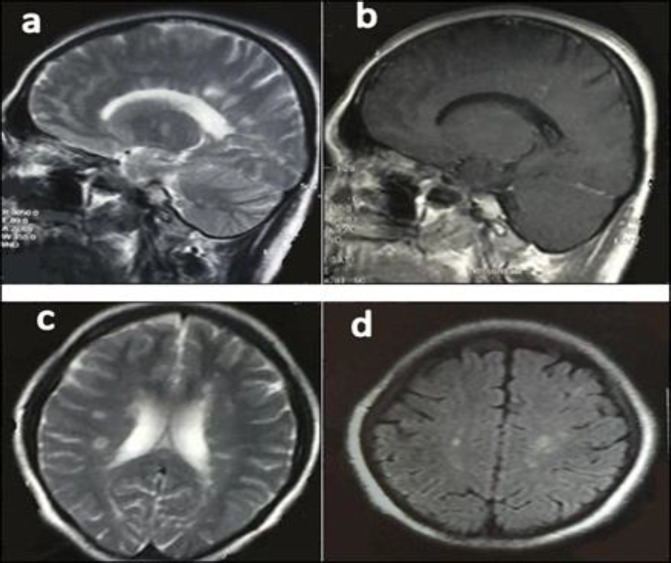
a) Sagittal T2 MRI, multiple hyperintense lesions involving periventricular white matter thalamus and cerebellum; b) Sagittal T1-weighted MRI showed enhancement of one periventricular lesion; c) and d) Axial T2 and FLAIR MRI, respectively; demonstrating multiple periventricular, centrum semioval, and juxtacortical lesions

The second EMG in 2011 showed a myopathic process pronounced in the proximal muscles. With a diagnosis of LGMD, rehabilitation, physical therapy, and stretching exercises were advised to improve her mobility and prevent disused atrophy or contracture deformities. In April 2015, she complained of acute left visual loss for four days with painful eye movements that lasted for several days. Her muscle strength had not changed for 5 years. On examination of her pupils, the left one had 2+ *relative afferent pupillary defects* (RAPD). Left visual acuity was finger counting from one meter with cecocentral scotoma in the visual field. Extraocular muscles and other cranial nerves were intact. Ophthalmologic consultation revealed no bulbar ophthalmic problems. For further evaluation, we planned a brain and optic pathway MRI that excluded compressive lesions of the optic pathway and disclosed multiple periventricular, centrum semiovale, corpus callosum, juxta cortical, and infra tentorial (brain stem and cerebellum) T2 hypersignal demyelinating plaques with multiple lesion enhancement (fig 3) that fulfilled the McDonald criteria for MS (3, 4).

From this, we prescribed intravenous methyl prednisolone (5 gr divided in 5 days) for the treatment of her optic neuritis. She responded significantly and her visual acuity ameliorated so that she could read from 30 cm. Vasculitis tests and anti-aquaporin 4 antibodies were negative. The final diagnosis was relapsing-remitting MS (RRMS). For long term treatment, we decided to put her on a glatiramer acetate daily injection.

## Discussion

This is the first report of LGMD in association with MS. Mutations in more than 50 loci have been known for LGMD. LGMD is distinct from x-linked muscular dystrophy by the autosomal pattern of inheritance, and from facioscapulohumeral and Emery muscular dystrophy by sparing the facial muscles. A proximal muscle involvement will become evident either in late childhood or early adulthood and is relatively benign in comparison with Duchenne dystrophy. Cardiac involvement occurs in some subtypes of the disease and mental functions are usually normal (5-8).

No definitive treatment exists for LGMD; however, management should be based for weight control, rehabilitation, physiotherapy, orthopedic surgery for skeletal deformities, and if respiratory failure occurs, then the use of respiratory aids and a mechanical ventilator is required (9-11). Although some reports suggest an association between myopathy and other neurological disorders, evidence is lacking to conclude this relationship. 

MS is another disabling neurologic disease, the most common autoimmune disorder affecting the central nervous system and is believed to occur as a result of genetics (including HLA DR15 and DQ56) and environmental factors (12). In recent years, the role of mitochondrial dysfunction in axonal loss degeneration in the progressive stage of MS has been investigated (13).

Some cases of MS and myopathy have been reported, for example, one case with centronuclear myopathy and RRMS (14). A 31-year-old woman with myotonic dystrophy type 2 presented with left hemihyperesthesia and mild spinal cord and brain stem symptoms whose paraclinical findings provided a diagnosis of RRMS (15). Two cases with mitochondrial myopathy and MS have been reported (16). The association of facioscapulohumeral muscular dystrophy and MS in a man with a history of bilateral pectoralis, proximal weakness, and atrophy of upper extremities since he was 16 years old showed left optic neuritis as the first presentation of MS at age 23 (17). A 27-year-old woman with progressive proximal lower limb weakness and increased liver function tests since her 20s with evidence of MS based on a brain MRI, cerebrospinal fluid, and visual evoked potential and in combination with an observation of periodic acid-Schiff (PAS) positive vacuoles on her muscle biopsy that was conclusive for adult onset Pompe disease with PPMS (18). The simultaneous occurrence of MS with myopathies may be incidental but there may be a genetic susceptibility for both diseases.

In conclusion the comorbidity between MS and others medical disorders is important for the treatment of this autoimmune disease. As the authors know this is the first report of MS in association with LGMD.
